# Associations of Dietary Patterns with Incident Depression: The Maastricht Study

**DOI:** 10.3390/nu13031034

**Published:** 2021-03-23

**Authors:** Vincenza Gianfredi, Annemarie Koster, Anna Odone, Andrea Amerio, Carlo Signorelli, Nicolaas C. Schaper, Hans Bosma, Sebastian Köhler, Pieter C. Dagnelie, Coen D.A. Stehouwer, Miranda T. Schram, Martien C.J.M. van Dongen, Simone J.P.M. Eussen

**Affiliations:** 1 CAPHRI Care and Public Health Research Institute, Maastricht University, 6200 MD Maastricht, The Netherlands; gianfredi.vincenza@hsr.it (V.G.); a.koster@maastrichtuniversity.nl (A.K.); n.schaper@mumc.nl (N.C.S.); hans.bosma@maastrichtuniversity.nl (H.B.); mcjm.vandongen@maastrichtuniversity.nl (M.C.J.M.v.D.); 2 CARIM School for Cardiovascular Diseases, Maastricht University, 6200 MD Maastricht, The Netherlands; dagnelie@maastrichtuniversity.nl (P.C.D.); cda.stehouwer@mumc.nl (C.D.A.S.); m.schram@maastrichtuniversity.nl (M.T.S.); 3 School of Medicine, Vita-Salute San Raffaele University, 20132 Milan, Italy; signorelli.carlo@hsr.it; 4 Department of Social Medicine, Maastricht University, 6200 MD Maastricht, The Netherlands; 5 Department Public Health, Experimental and Forensic Medicine, University of Pavia, 27100 Pavia, Italy; anna.odone@unipv.it; 6 Department of Neuroscience, Rehabilitation, Ophthalmology, Genetics, Maternal and Child Health (DINOGMI), Section of Psychiatry, University of Genoa, 16126 Genoa, Italy; andrea.amerio@unige.it; 7 IRCCS Ospedale Policlinico San Martino, 16132 Genoa, Italy; 8 Mood Disorders Program, Tufts Medical Center, Boston, MA 02111, USA; 9 Department of Internal Medicine, Maastricht University, 6200 MD Maastricht, The Netherlands; 10 Department of Psychiatry and Neuropsychology, Maastricht University, 6200 MD Maastricht, The Netherlands; s.koehler@maastrichtuniversity.nl; 11 MHeNS School for Mental Health and Neuroscience, Maastricht University, 6200 MD Maastricht, The Netherlands; 12 Heart and Vascular Center, Maastricht University Medical Center+, 6200 MD Maastricht, The Netherlands; 13 Department of Epidemiology, Maastricht University, 6200 MD Maastricht, The Netherlands

**Keywords:** Dutch Healthy Diet score, Mediterranean diet, dietary approaches to stop hypertension, depressive symptoms, major depressive disorder, prospective cohort study

## Abstract

Our aim was to assess the association between *a priori* defined dietary patterns and incident depressive symptoms. We used data from The Maastricht Study, a population-based cohort study (*n* = 2646, mean (SD) age 59.9 (8.0) years, 49.5% women; 15,188 person-years of follow-up). Level of adherence to the Dutch Healthy Diet (DHD), Mediterranean Diet, and Dietary Approaches To Stop Hypertension (DASH) were derived from a validated Food Frequency Questionnaire. Depressive symptoms were assessed at baseline and annually over seven-year-follow-up (using the 9-item Patient Health Questionnaire). We used Cox proportional hazards regression analyses to assess the association between dietary patterns and depressive symptoms. One standard deviation (SD) higher adherence in the DHD and DASH was associated with a lower hazard ratio (HR) of depressive symptoms with HRs (95%CI) of 0.78 (0.69–0.89) and 0.87 (0.77–0.98), respectively, after adjustment for sociodemographic and cardiovascular risk factors. After further adjustment for lifestyle factors, the HR per one SD higher DHD was 0.83 (0.73–0.96), whereas adherence to Mediterranean and DASH diets was not associated with incident depressive symptoms. Higher adherence to the DHD lowered risk of incident depressive symptoms. Adherence to healthy diet could be an effective non-pharmacological preventive measure to reduce the incidence of depression.

## 1. Introduction

Depression is a mental disorder that ranks fourth among the most common causes of disease burden worldwide, and according to the World Health Organization, it is expected to rank first in the 2030s [1]. Moreover, depression is highly recurrent, demanding for both more effective treatments and preventive actions [1].

Previous studies showed that higher dietary intake of specific nutrients, as for instance, folate, vitamin B12, vitamin D, n-3 fatty acids, and zinc might reduce the risk of mental health disorders, particularly depression [2]. However, instead of isolated nutrients, people rather consume meals consisting of mixed food groups, which in turn are sources of various nutrients. Hence, studies to date investigated associations of dietary patterns with depression. The majority of studies focused on adherence to the Mediterranean diet and recent meta-analyses concluded that a high adherence to this diet was generally associated with lower odds of having prevalent depression [3,4], while adherence to this dietary pattern was associated with lower risk of incident depression in some [4], but not all studies [3]. Similarly, adherence to the Dietary Approach to Stop Hypertension (DASH) diet, which was firstly developed to reduce the risk of hypertension [5], was associated with lower prevalent (defined as number of depressed cases within the population) [6] and incident depression (defined as new diagnosis of depression over time) [4,7]. Recently, higher adherence to the Dutch Healthy Diet (DHD), which specifically reflects adherence to the Dutch dietary guidelines [8], was cross-sectionally associated with lower risk of prevalent depression in diabetes patients [9].

Considering that the majority of available studies were cross-sectional analyses [3,6,10] and that existing evidence yielded mixed results, more prospective studies with high methodological quality are needed to confirm the validity of available evidence. Therefore, the aim of this study was to assess the prospective association between adherence to three a priori defined dietary patterns and incident depression in an adult population. In this analysis we included adherence to Mediterranean and DASH diets in order to compare results with the available literature, and additionally include adherence to the DHD, a dietary pattern that specifically reflects adherence to the dietary guidelines of the general Dutch population (our study population).

## 2. Materials and Methods

### 2.1. Study Population and Design

**Figure 1:**
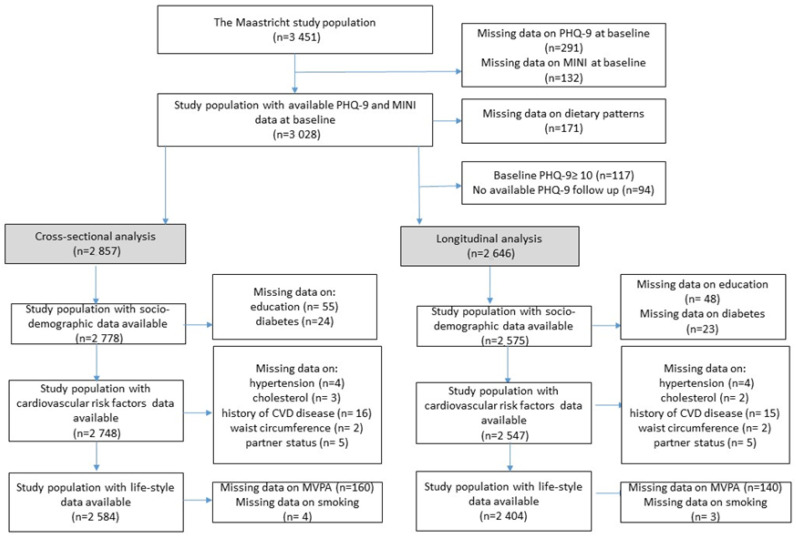
Flowchart of the study population. MINI: Mini-International Neuropsychiatric Interview; PHQ-9: 9-item Patient Health Questionnaire; CVD: Cardiovascular diseases; MVPA: Moderate-to-vigorous Physical Activity.

We used data from The Maastricht Study, a population-based observational prospective cohort study enriched with patients with type 2 diabetes mellitus (T2DM). Details about the rationale and the methodology of this study were published elsewhere [11]. In brief, the study focused on the aetiology, pathophysiology, complications, and comorbidities of T2DM and other chronic diseases; it was characterized by an extensive phenotyping approach. Eligible participants were all individuals aged 40–75 years and living in the southern part of the Netherlands. Participants were recruited through mass media campaigns and from the municipal registries and the regional Diabetes Patient Registry via mailings. Recruitment was stratified according to known T2DM status, with an oversampling of individuals with T2DM, for reasons of efficiency. The present study includes longitudinal data from the first 3451 participants, who completed the baseline survey between November 2010 and September 2013. The baseline examinations of each participant were performed within a time window of three months. Annual follow-up data were available in 91.4 (year 1), 85.4 (year 2), 79.6 (year 3), 71.8 (year 4), 76.6 (year 5), 65.9 (year 6), and 28.2% (year 7) of the participants. The relatively low number of participants for years 6 and 7 is because the follow-up measurement is still ongoing. Figure 1 shows the flowchart of the study population. Data on depression (*n* = 423 missing) and dietary patterns (*n* = 171 with implausible energy intake) at baseline were available in *n* = 2857 participants. For longitudinal analysis, participants with depression at baseline (*n* = 117), and without any follow-up data on depression (*n* = 94) were also excluded, resulting in a final sample size of 2646 participants (of whom 652 participants had T2DM, see below).

### 2.2. Assessment of Depression at Baseline

At baseline, severity and presence of depressive symptoms were assessed by means of a validated Dutch version of the 9-item Patient Health Questionnaire (PHQ-9) [12]. The PHQ-9 is a self-administered questionnaire based on the DSM-IV criteria for major depressive disorder (MDD). The PHQ-9 measures both cognitive symptoms of depression and somatic symptoms of depression [13]. Each item is rated on a 4-point ordinal scale from 0 = “not at all” to 3 = “nearly all of the time”. The total score ranges between 0 to 27. When one or two items were missing, the total-score was calculated as 9× (total points/(9−number of missing items)) and rounded to the nearest integer. A pre-defined cut-off score of ≥10 was used as a dichotomous scoring system for defining clinically relevant depressive symptoms [14].

At baseline, prevalent and lifetime major depressive disorder (MDD) was assessed by the Mini-International Neuropsychiatric Interview (MINI) [15]. The MINI is a short diagnostic structured interview, used to assess the presence of minor or major depressive disorder in the preceding two weeks according to the DSM-IV (Diagnostic and Statistical Manual of Mental Disorders, Fourth Edition). MDD was diagnosed if participants had five symptoms, of which at least one was a core symptom plus other symptoms of depression. Lifetime history of MDD was assessed by asking for the presence of symptoms during a minimum of two weeks in their lifetime.

### 2.3. Assessment of Depression during Follow-Up

Incident depressive symptoms were assessed by use of the PHQ-9 questionnaire annually during 7 years follow up. Incident clinically relevant depressive symptoms were defined as no depression at baseline (PHQ-9 < 10) and presence of clinically relevant depressive symptoms on at least one follow-up moment (PHQ-9 ≥ 10).

### 2.4. Assessment of Dietary Patterns

Diet was assessed at baseline using a validated, self-administered FFQ [16]. The FFQ assessed the frequency and amount of consumed foods from 23 product groups comprising 253 food items, with a 1-year reference period. Frequency questions used an answer model with 11 options, from not at all to daily (7 days/week). Each of the frequency questions was combined with an amount (quantity) question, using an answer model with fourteen standard household servings, from <1/day to >12/day. More details on the development and the validity of this FFQ were published elsewhere [17]. Intake of total energy and nutrients were calculated using the Dutch Food Composition Database (NEVO) [18].

#### 2.4.1. Dutch Healthy Diet Score

The Dutch Healthy Diet score 2015 (DHD-score) consists of fifteen components representing the fifteen food-based Dutch dietary guidelines updated in 2015 [8]. For each food component, the score ranged between 0 and 10 points. For a healthy food component (adequacy component; i.e., vegetables, fruits, wholegrain products, legumes, nuts, fish, tea), intake equal to or higher than a cut-off value, specified according to the dietary guidelines, the maximum score was given, whilst for consumption below that cut-off value the score was calculated by means of linear interpolation between threshold value (often 0; score = 0) and cut-off value (score = 10). A detailed description of the operationalization is provided elsewhere [8]: Cut-off values for healthy components were 200 g/day for vegetables, 200 g/day for fruits, 90 g of whole grain products/day (max 5 points) in combination with a ratio of whole grains to refined grains (max 5 points;, 10 g/d for legumes, 15 g/d for nuts, 15 g/d for fish with a maximum for lean fish of 4 g/d), and 450 g/d for tea. For an unhealthy food component (moderation component; i.e., dairy, fats and oils, coffee, red meat, processed meat, sweetened beverages and fruit juices, alcohol, and salt), the maximum score was assigned if the consumption was equal to or below a specific cut-off value, whereas for consumption higher than that cut-off value the score was calculated by means of linear interpolation between the cut-off value (score = 10) and the threshold value (score = 0). The unhealthy food products included fats and oils (the solid/liquid fat (cooking fat, bread smear)) intake ratio, with a threshold (0 points) of 1.67 (reflecting the 85th percentile score of the Dutch population) and a cut-off value (10 points) of 0.08 (reflecting the 15th percentile score of the Dutch population), red meat (cut-off: 45 g/d; threshold: 100 g/d), processed meat (cut-off: 0 g/d; threshold: 50 g/d), sweetened beverages and fruit juices (cut-off: 0 g/d; threshold: 250 g/d), sodium (cut-off: 1.9 g/d; threshold 3.8 g Na), and alcohol (cut-off: 10 g ethanol/d; threshold: 20 and 30 g ethanol/d, for women and men, respectively). The consumption of dairy products was considered to be healthy, but only at moderate intake levels (300–450 g/d, including 40 g of cheese at maximum). For this ‘optimum’ component, separate cut-off and threshold levels hold for the below optimum (cut-off: 300 g/d; threshold: 0 g/day) and the above optimum (cut-off: 450 g/d; threshold: 750 g/d) levels, respectively. Coffee consumption is a qualitative component based on type of coffee (filtered vs. unfiltered). However, this information was not part of the FFQ and hence could not be included in our calculation of the DHD-score. For this reason, the sum of the scores for every food component resulted in the DHD-score overall score with a minimum of 0 and a maximum of 140 [8].

#### 2.4.2. Mediterranean Diet and Dietary Approaches to Stop Hypertension Scores

The Mediterranean diet score is based on 9 food groups (vegetables, legumes, fruits and nuts, fish, cereals, dairy, meat, ratio (MUFA+PUFA)/SFA, and ethanol), with sex-specific medians of intakes as cut-off values. The median intake for food groups were derived from the FFQ. For each food component, a score of 0 or 1 was assigned. For healthy components (vegetables, legumes, fruits and nuts, fish, cereals, ratio (MUFA+PUFA)/SFA) a score of 0 was assigned for intake below the median, whilst a score of 1 was granted for intake higher or equal to the median. For unhealthy food components (dairy, meat), the scores were inverted (1 for intake below the median, 0 for intake above the median). Regarding alcohol consumption, a score of 1 was allocated if consumption was between 10 and 50 g/d for males and between 5 and 25 g/d for females, and a score of 0 for any other amount of alcohol consumption. The sum of the scores for every food group results in the overall Mediterranean diet score (minimum 0, maximum 9) [19]. The DASH score is based on 8 food groups and uses sex-specific quintiles as cut-off values. For each food component the score can range between 1 and 5, assigned proportionally to the intake level. Both for Mediterranean and DASH, a higher intake of healthy food components (vegetables, fruits, nuts and legumes, wholegrain products, low-fat dairy) corresponds to higher scores, and vice versa for unhealthy food components (red and processed meat, sugar sweetened beverages, and sodium intake). The sum of the scores for each food group results in the overall DASH score (minimum 8, maximum 40) [5]. Higher scores on the DHD-score [8], the Mediterranean diet [19] and the DASH diet [5] reflect better adherence to a healthy dietary patterns.

### 2.5. Covariates

The following variables, measured at baseline, were considered as potential confounders. Self-reported data comprised sex, age, level of education (low = no education/primary education/lower vocational education, medium = intermediate vocational education/higher secondary education/higher vocational education, high = higher professional education/university education), partner status (yes/no), history of cardiovascular disease (Rose Questionnaire), smoking status (current, former, and never smokers), moderate-to-vigorous physical activity (Community Healthy Activities Model Program for Seniors (CHAMPS) questionnaire), and energy intake. Covariates obtained from a clinical examination included, height, weight, waist circumference, office blood pressure, plasma glucose levels, and plasma lipid profile. Moderate-to-vigorous physical activity included activities as fast walking, fast cycling, heavy gardening, heavy household work, jogging/running, swimming, tennis, team sport, and intensive exercise. Energy intake was expressed in kcal/day and measured by a validated Food Frequency Questionnaire [16]. Participants with implausible energy intake (men: energy intake (kcal) <800 kcal or >4200 kcal; women: energy intake (kcal) <500 kcal or >3500 kcal) were excluded. We measured, height, weight, waist circumference, office blood pressure, plasma glucose levels, and plasma lipid profile. Type 2 diabetes status was defined by a standardized 2-h 75-g oral glucose tolerance test after an overnight fast, performed at the research center, and use of antidiabetic medication as previously described [11]. Medication use was assessed in a medication interview where generic name, dose, and frequency were registered.

### 2.6. Statistical Analysis

Descriptive characteristics of the study population were presented as mean and standard deviation (SD) for continuous variables or numbers and percentages for categorical variables. To assess differences between participants with and without depressive symptoms, we performed chi-square, and analysis of variance (ANOVA) tests, as appropriate. All dietary pattern scores were analyzed as standardized score and categorized into tertiles. In addition, for the Mediterranean diet we also applied predefined literature-based cut-off values [19]. We conducted multivariable logistic regression analyses to examine the association of dietary patterns with presence of clinically relevant depressive symptoms and MDD at baseline. Using Cox proportional hazards regression analyses, we assessed the association between dietary patterns and incident clinically relevant depressive symptoms over time. Cox proportional hazard assumption was verified by visually inspecting the Kaplan–Meier curves, and it was not violated. For each dietary pattern, hazard ratios (HRs) and 95% confidence intervals (95% CIs) were estimated. We adjusted for several covariates in the analyses. Model 1 was adjusted for age, sex, level of education, and diabetes status (model 1). Additional adjustments were made for hypertension, total cholesterol and High-Density Lipoprotein (HDL) cholesterol, history of cardiovascular diseases (CVD), waist circumference, and partner status (model 2). Finally, the associations were adjusted for lifestyle risk factors, in particular physical activity, smoking, and energy intake (model 3). To test the linearity of the association between dietary score and state of depression, a test of linear trend was conducted over the tertiles, using the median value in each tertiles. Sensitivity analyses encompassed further adjustment for (1) alcohol intake, (2) occupational status, (3) antidepressant drugs use; (4) MDD at baseline, in addition, we excluded individuals (5) that use antidepressant drugs at baseline, (6) participants with MDD at baseline, and (7) participants with lifetime MDD. Moreover, we conducted a strict analysis (8) using a group with maximum 2 missing follow-up measurements for the longitudinal analyses. In addition, as depression is known to differ between men and women [20], we performed an interaction analysis for sex. Lastly, as The Maastricht Study had by design an oversampling of participants with diabetes, we evaluated the potential interaction of diabetes status with the diet-depression association. All analyses were conducted using IBM SPSS software version 21.0 (IBM Corp., Armonk, NY, USA). Associations with *p* < 0.05 in two-sided tests were considered to be statistically significant. Since participants of the cohort study were followed for different lengths of time (as for instance different recruitment time or a different period free of depressive symptoms), it was important to estimate the actual time-at-risk that all participants contributed to the study. Participants could contribute to the calculation of person-years until depressive symptoms were identified, participants died, or were lost to follow up.

## 3. Results

### 3.1. Descriptive Characteristics of the Population

Table 1 shows the baseline characteristics of the study population according to incident (yes/no) clinically relevant depressive symptoms (PHQ-9 questionnaire ≥ 10). Participants had a mean (SD) age of 59.9 (8.0) years and 49.5% were women. During 15,188 person-years of follow-up, 315 (11.9%) participants developed clinically relevant depressive symptoms, which yielded an incidence rate of 20.74 cases per 1000 person-years. General characteristics of the study population at baseline, stratified by presence (yes/no) of prevalent clinically relevant depressive symptoms and MDD (cross-sectional analysis) are shown in Supplementary Table S1. Participants with MDD, prevalent clinically relevant depressive symptoms, and who incidentally developed these symptoms had a lower level of education, were more often current smokers, consumed less alcohol, had a larger waist circumference, had more often hypertension, had higher total cholesterol-to-HDL cholesterol ratio, and more often a history of cardiovascular diseases, compared to participants who did not develop clinically relevant depressive symptoms.

### 3.2. Dietary Patterns and Incident and Prevalent Depressive Symptoms

Table 2 shows the associations of dietary patterns with incident clinically relevant depressive symptoms. When the standardized scores were analyzed, the Mediterranean diet, DASH, and DHD–scores were statistically significantly associated with incident clinically relevant depressive symptoms in model 1. After further adjustment for cardiovascular risk factors, associations remained significant with HR (95%CI) per higher SD in dietary score of 0.78 (0.69–0.89) for the DHD, and 0.87 (0.77–0.98) for the DASH. In line with this, individuals in the highest tertile compared to the lowest tertile for adherence to DHD and DASH had 34 and 29% lower risk of clinically relevant depressive symptoms, with HR (95%CI) of 0.66 (0.49–0.90) for DHD and 0.71 (0.52–0.97) for DASH (*p* for trend <0.05), respectively. After further adjustment for the lifestyle factors smoking, physical activity, and energy intake, only the DHD-score remained significantly associated with incident clinically relevant depressive symptoms, with a HR per SD of 0.83 (0.73–0.96). None of the dietary pattern scores were associated with prevalent clinically relevant depressive symptoms (*n* = 117) and MDD (*n* = 89) (supplementary Table S2).

### 3.3. Sensitivity Analysis

To a large extent, similar associations were found after additional adjustment for (1) alcohol intake, (2) occupational status, (3) antidepressant drugs use, and (4) presence of MDD at baseline. In addition, exclusion of (5) participants with antidepressant use at baseline (*n* = 125), (6) participants with MDD at baseline (*n* = 33), (7) participants with lifetime MDD (*n* = 706), and (8) allowing at maximum 2 missing follow-up measurements for the longitudinal analyses resulted in essentially similar associations. However, after exclusion of participants with lifetime MDD, associations between dietary patterns and incident clinically relevant depressive symptoms became slightly stronger, with HR (95%CI) per SD of 0.73 (0.60–0.90) for DHD, of 0.83 (0.69–1.01) for DASH, and of 0.79 (0.65–0.96) for Mediterranean diet (Supplementary Table S3). In addition, we tested for effect modification by sex and diabetes status; these factors did not modify the associations between diet and incident clinically relevant depressive symptoms (*p* for interaction >0.05, data not shown).

## 4. Discussion

We investigated the association of *a priori*-defined adherence to the DHD and to commonly investigated Mediterranean and DASH dietary patterns with incident clinically relevant depressive symptoms. Models adjusted for demographic, cardiovascular, and lifestyle factors revealed that a higher adherence to the DHD was associated with lower risk of developing clinically relevant depressive symptoms over a median follow-up period of 6.1 years, but adherence to the Mediterranean and DASH diets was not associated with incident clinically relevant depressive symptoms.

Results from the current study are in line with the observation that a healthy diet is associated with lower risk of depression [4,21]. However, when different healthy diets are compared—as in our study—seemingly different results were observed. Two meta-analyses from 2019 reported contrasting conclusions on the Mediterranean Diet, one showing that a higher adherence was associated with a lower risk of clinically relevant depressive symptoms during follow-up with an OR of 0.88 (0.80–0.96) [4], whereas the other meta-analysis did not observe an association with depression risk (HR 0.95 (0.79–1.16)) [3]. In an another study among elderly subjects, DASH, but not a Mediterranean Diet, was associated with a higher depression risk over time [21]. These seemingly contrasting results can probably be explained by the use of different scores to quantify adherence to a Mediterranean diet, differences in population characteristics, as well as differences in the duration of follow-up and the variables considered as confounders.

Even though only DHD was significantly associated with incident depression in the present study, risk estimates for Mediterranean and DASH diet revealed similar directions of the associations. These differences in effect sizes between the dietary patterns could be explained by differences in operationalization procedures for each dietary pattern score, in the present and previous studies [3,4,21]. Since there is no clear consensus in the literature on the various algorithms to compute the Mediterranean and DASH diet, we operationalized adherence according to the original publications for both Mediterranean diet [19] and DASH diet [5]. Adherence to the DHD [8], the Mediterranean Diet [19], and the DASH dietary patterns were based on 14, 9, and 8 food groups, respectively. Apart from the number of food groups, the composition of these groups differs between the three dietary patterns. For instance, DHD and DASH account for the consumption of wholegrain cereals instead of cereals in general, as is the case for the Mediterranean Diet. Similarly, DHD and DASH consider fruit consumption as a separate component, while the Mediterranean Diet combines fruits with nuts. In the DHD, nuts are considered as a stand-alone food group but are combined with legumes in DASH. In this respect, the DHD is, in comparison to the DASH or Mediterranean Diet, the most comprehensive detailed dietary score, which potentially has greater sensitivity to detect potential diet-disease associations. The apparent null finding for the Mediterranean diet and depression risk may be explained by its operationalization procedure, and by the fact that most Dutch people do not adhere to a Mediterranean food culture. Nonetheless, all three dietary patterns studied included key components with beneficial health effects such as fruits, vegetables, and fish as well as components with potential deleterious health effects such as red and processed meats. The DHD, is particularly relevant for the investigated study population [8]—since it is a reflection of national Dutch guidelines—however, we believe that results of the current study could be generalized towards other populations as well. To the best of our knowledge, so far only one cross-sectional study assessed the association between higher adherence to DHD and lower risk of depression among patients with diabetes mellitus, with a smaller sample size, and with a less extensive FFQ that enabled to operationalize 12 out of 15 components of the original score [8].

### 4.1. Potential Biological Mechanisms

The biological mechanisms underlying the association between diet and depression are not completely understood. Studies in this area are limited by the fact that a dietary pattern consists of multiple food groups and components, which can have diverse neuro-psychological effects, and which could possibly interact with each other. Evidence has pointed towards the involvement of food components in the monoamine synthesis, inflammation processes, regulation of hypothalamic–pituitary–adrenal axis (HPA), and neurogenesis [22]. A higher consumption of refined and processed foods, as well as high-fat and high-sugar products is associated with higher levels of inflammation and higher risk of depression [23,24]. At the same time, inflammation can be one of the factors implicated in the microvascular dysfunction, which in turn can be responsible for the alterations in brain regional blood flow that were shown to be a risk factor for depression [25].

Recently, we reported that, using data from The Maastricht Study, various markers of endothelial dysfunction were associated with incident clinically relevant depressive symptoms [26]. Moreover, microvascular dysfunction in this cohort was related to hyperglycemia [27], glycation [28], and alterations in circulating lipids [29]. All these metabolic changes can be the result of changes in diet, among other lifestyle factors, and contribute to depression via microvascular dysfunction. Nevertheless, microvascular dysfunctions can be also a consequence of other factors such as smoking, obesity, hypertension [25], mental stress [30], and physical activity, which is also associated with lower risk of depression [31].

### 4.2. Strengths and Limitations

Major strengths of this study are the large sample size and the longitudinal study design, with a median follow up period of 6.1 years. Moreover, results were controlled for a comprehensive range of confounders including sociodemographic characteristics, CVD risk factors and lifestyle factors. The deep-phenotyping assessment in the current study enabled us to conduct multiple sensitivity analyses, which revealed essentially similar associations, which further strengthened the validity of our study findings. In this regard, the broad range of sensitivity analyses supports the idea of a causal association between dietary patterns and risk of incident depression.

Another strength is the validated, comprehensive FFQ used to estimate the level of adherence to each of the three different dietary patterns. With 253 food items included, The Maastricht Study FFQ is currently one of the most comprehensive FFQs, and this large number of food items facilitates reliable estimations of intake of specific food groups included in the dietary patterns. In addition, the use of evidence-based threshold values in the DHD-score allows comparison of results with future studies and generalizability towards other populations.

Although the PHQ-9 questionnaire is a validated screening instrument to measure depressive symptoms, suggestive of depression, it is not a diagnostic tool for MDD. However, as the PHQ-9 has a high sensitivity and specificity compared to the psychiatric interview as a reference standard, not necessarily equated with MDD [14]. Considering similar results were obtained in sensitivity analyses, we do not expect that this low degree of misclassification would affect our results. Moreover, there could have been selection and/or attrition bias, which is inherent to prospective population-based studies; individuals with more severe depressive symptoms or with greater comorbidity may have been more likely not to participate or to withdraw. However, this is also reflected in the relatively low number of prevalent cases at baseline, and this might have led to an underestimation of effect sizes of cross-sectional analyses. Lastly, depressive individuals often suffer from low self-efficacy, which may affect their ability to comply with medications and healthier lifestyles, resulting in a negative vicious circle. This aspect might lead to possible reverse causation. However, the longitudinal approach used in this study, where we excluded individuals with depressive symptoms at baseline, limits this risk of bias. In addition, in sensitivity analyses we excluded individuals with MDD at baseline and those using anti-depressant drugs, which did not materially change the results. Future studies may consider including repeated dietary assessment to capture dietary changes. In the present study, repeated dietary assessment was not available yet within the 6.1 year follow-up period.

## 5. Conclusions

In conclusion, in this middle-aged Caucasian population, a higher adherence to the DHD, using evidence-based threshold values for individual components, was statistically significantly associated with lower risk of incident depression during a seven years of follow-up. Intervention studies still need to confirm efficacy of healthy dietary patterns to prevent or reduce depressive symptoms. Evidence so far suggested that promoting healthy dietary patterns into daily life could be an inexpensive non-pharmacological strategy that contributes to the prevention of depression at a population level. Supporting health education intervention promoting healthy eating seems particularly relevant in light of the high burden of depression worldwide.

## Figures and Tables

**Table 1:** Baseline characteristics of the study population stratified by incident clinically relevant depressive symptoms.

Characteristic	No Incident Depressive Symptoms (PHQ-9 < 10 during Follow-Up) *n* = 2331	Incident Clinically Relevant Depressive Symptoms (PHQ-9 ≥ 10) during Follow-Up *n* = 315	*p*-Value
**Sex (women)**	1154 (49.5)	157 (49.8)	0.911
**Age (years)**	59.92 ± 8.02	59.78 ± 8.35	0.767
**Education**			0.000
**Low**	680 (29.7)	123 (39.9)	
**Medium**	650 (28.4)	98 (31.8)	
**High**	960 (41.9)	87 (28.8)	
**Smoking**			0.000
**Never**	850 (36.9)	100 (32.2)	
**Former**	1223 (53.2)	153 (49.2)	
**Current**	228 (9.9)	58 (18.6)	
Waist circumference (cm) *	94.55 ± 12.96	99.46 ± 14.91	0.000
**BMI (kg/m^2^)**	26.64 (4.23)	28.28 (4.93)	0.000
**Hypertension**	1267 (54.4)	202 (64.1)	0.001
Total cholesterol-to-HDL cholesterol ratio *	3.63 ± 1.15	3.77 ± 1.24	0.037
**History of CVD**	350 (15.3)	72 (23.3)	0.000
**Diabetes**			0.000
**No diabetes**	1422 (61.5)	141 (45.8)	
**Pre-diabetes**	362 (15.7)	44 (14.3)	
**T2DM**	529 (22.9)	123 (39.9)	
MVPA (hours/week) *	5.76 ± 4.33	4.45 ± 4.05	0.000
**Having a partner (yes)**	1975 (85.8)	257 (83.4)	0.262
**Depression**			
Depression score at baseline (PHQ-9 score) *	1.95 ± 20.7	4.25 ± 2.72	0.000
**Major depressive disorder at baseline (MINI)**	21 (0.9)	18 (5.7)	0.000
**Use of antidepressants at baseline**	103 (4.4)	45 (14.3)	0.000
Diet *			
**Energy intake (Kcal)**	2177 ± 595	2232 ± 630	0.125
**Protein total (g/day)**	85.6 ± 22.5	87.4 ± 25.0	0.196
**Carbohydrates total (g/day)**	231.8 ± 68.1	238.0 ± 74.6	0.135
**Fat total (g/day)**	83.9 ± 30.7	88.3 ± 32.1	0.018
**Fatty acids total saturated (g/day)**	29.4 ± 11.9	31.3 ± 12.4	0.011
**Fatty acids total monounsaturated (d/day)**	29.6 ± 11.3	30.8 ± 11.2	0.059
**Fatty acids total polyunsaturated (g/day)**	17.6 ± 7.7	18.6 ± 8.4	0.044
**Alcohol intake (g/day)**	12.8 ± 13.8	10.5 ± 14.5	0.003
**DHD, (range 0–140)**	84.37 ± 14.50	79.68 ± 15.11	0.000
**Mediterranean Diet Score, (range 0–9)**	4.61 ± 1.64	4.32 ± 1.60	0.003
**DASH score, (range 8–40)**	24.24 ± 4.51	23.11 ± 4.37	0.000

* Results are presented as mean ± SD or *n* (%). CVD: Cardiovascular diseases; DASH: Dietary Approaches To Stop Hypertension; DHD: Dutch Health Diet; HDL: High-Density Lipoprotein; MVPA: Moderate-to-vigorous physical activity. MINI: Mini-International Neuropsychiatric Interview; PHQ-9: 9-item Patient Health Questionnaire.

**Table 2:** Longitudinal association of dietary patterns with incident clinically relevant depressive symptoms during 7-years of follow-up (median 6.1 years).

Incident Clinically Relevant Depressive Symptoms (PHQ-9 ≥ 10)	Incident Rate Per 1000 Person Years *n* = 2646	Model 1 HR (95% CI) *n* = 315/2273	Model 2 HR (95% CI) *n* = 298/2249	Model 3 HR (95% CI) *n* = 280/2124
**DHD-score**				
Standardized score *		0.75 (0.67–0.85)	0.78 (0.69–0.89)	0.83 (0.73–0.96)
**Tertiles**				
**Low (≤77.27)**	[*n* = 139] 9.15	Ref	Ref	Ref
**Medium (77.27–90.43)**	[*n* = 96] 6.32	0.70 (0.53–0.91)	0.74 (0.56–0.97)	0.83 (0.63–1.12)
**High(>90.43)**	[*n* = 80] 5.27	0.61 (0.45–0.82)	0.66 (0.49–0.90)	0.77 (0.55–1.06)
**Linear trend *p*-value +**		0.006	0.031	0.246
**Mediterranean Diet Score**				
Standardized score *		0.87 (0.77–0.98)	0.89 (0.79–1.01)	0.92 (0.81–1.05)
Cut-off **				
**Low (0–3)**	[*n* = 94] 6.18	Ref	Ref	Ref
**Medium (4–6)**	[*n* = 72] 4.74	0.90 (0.66–1.23)	0.93 0.68–1.28)	0.94 (0.68–1.31)
**High (6–9)**	[*n* = 149] 9.81	0.82 (0.63–1.07)	0.88 (0.67–1.16)	0.96 (0.72–1.28)
**Linear trend *p*-value +**		0.354	0.570	0.758
DASH score *				
**Standardized score**		0.85 (0.75–0.95)	0.87 (0.77–0.98)	0.95 (0.83–1.07)
**Tertiles**				
**Low (≤22)**	[*n* = 139] 9.15	Ref	Ref	Ref
**Medium (22–26)**	[*n* = 110] 7.24	0.94 (0.73–1.22)	0.97 (0.75–1.26)	1.06 (0.81–1.39)
**High (>26)**	[*n* = 66] 4.35	0.66 (0.49–0.90)	0.71 (0.52–0.97)	0.83 (0.60–1.15)
**Linear trend *p*-value +**		0.024	0.076	0.477

* Standard deviations for DHD, Mediterranean and DASH diet scores were 14.7, 1.64, and 4.5, respectively. ** Based on literature, Trichopoulou A. et al. [19]. + Based on median. Model 1 adjusted for socio-demographic characteristics (age, sex, level of education) and diabetes status; Model 2 additional adjustment for cardiovascular risk factors: history of CVD, hypertension, total cholesterol and HDL cholesterol, waist circumference) and partner status; Model 3 additional adjustment for lifestyle factors (MVPA, smoking, and energy intake). (*n* = cases/non cases), [*n* = cases].

## Data Availability

The data that support the findings of this study are available from the corresponding author, Simone Eussen, upon reasonable request.

## Supplementary Materials

The following are available online at https://www.mdpi.com/2072-6643/13/3/1034/s1, Table S1: Baseline characteristics of the study population stratified for prevalent depression; Table S2: Cross-sectional association of dietary patterns with prevalent clinically relevant depressive symptoms and major depressive disorder; Table S3: Sensitivity analysis of the association of dietary patterns with incident clinically relevant depressive symptoms during 7-years of follow-up (median 6.1 years) additionally adjusted.
