# Supplementary material for: Associations of Dietary Patterns with Incident Depression: The Maastricht Study
Source: Nutrients. 2021 Mar 23;13(3):1034. doi: 10.3390/nu13031034 (PMC8004955; doi:10.3390/nu13031034)
Supplement: Supplementary file 1 [file nutrients-13-01034-s001.pdf]

nutrients

MDPI

Supplementary

Associations of Dietary Patterns with Incident Depression: the Maastricht Study

Vincenza Gianfredi [1,2,3], Annemarie Koster [1,4], Anna Odone [5], Andrea Amerio [6,7,8], Carlo Signorelli [3], Nicolaas C. Schaper [1,2,9], Hans Bosma [1,4], Sebastian Köhler [10,11], Pieter C. Dagnelie [2,9], Coen D. A. Stehouwer [2,9], Miranda T. Schram [2,9,11,12], Martien C. J. M. van Dongen [1,13] and Simone J. P. M. Eussen [2,13,*]

Table S1. Baseline characteristics of the study population stratified for prevalent depression.

Characteristic	Total Population (n = 2,857)	No Depression at Baseline (PHQ-9 < 10 and No MDD (MINI)) (n = 2,699)	Prevalent Clinically Relevant Depressive Symptoms (PHQ-9 ≥ 10) (n = 117 Cases)	Prevalent MDD (MINI) (n = 89 Cases)
Sex (women)	1,431 (50.1)	1,342 (49.7)	72 (61.5)	45 (50.6)
Age (years)	59.7 ± 8.15	59.86 ± 8.11	56.01 ± 7.73	58.73 ± 8.37
Education				
Low	894 (31.9)	829 (31.3)	45 (39.8)	40 (47.1)
Medium	802 (28.6)	754 (28.5)	36 (31.9)	26 (30.6)
High	1,106 (39.5)	1,067 (40.3)	32 (28.3)	19 (22.4)
Smoking				
Never	1,009 (35.8)	970 (36.4)	28 (24.8)	20 (23.0)
Former	1,469 (52.1)	1,392 (52.2)	54 (47.8)	49 (56.3)
Current	341 (12.1)	303 (11.4)	31 (27.4)	18 (20.7)
Waist circumference (cm)	95.52 ± 13.64	95.14 ± 13.31	102.05 ± 18.40	102.60 ± 17.48
BMI (kg/m²)	26.99 ± 4.50	26.85 ± 4.38	29.42 ± 6.13	29.42 ± 5.92
Hypertension	1,593 (55.8)	1,494 (55.4)	73 (62.4)	60 (67.4)
Total cholesterol-to-HDL cholesterol ratio	3.66 ± 1.17	3.64 ± 1.15	3.84 ± 1.45	3.98 ± 1.37
History of CVD	464 (16.5)	427 (16.1)	27 (23.7)	20 (22.7)
Diabetes				
No diabetes	1,654 (58.4)	1,587 (59.3)	51 (44.0)	38 (43.2)
Pre-diabetes	434 (15.3)	413 (15.4)	16 (13.8)	11 (12.5)
T2DM	769 (26.2)	675 (25.2)	49 (42.2)	39 (44.3)
MVPA (hours/week)	5.50 ± 4.33	5.59 ± 4.3	4.03 ± 4.47	3.64 ± 3.13
Having a partner (yes)	2,381 (84.6)	2,269 (85.2)	82 (71.9)	67 (77.0)
**Depression**				
Depression score at baseline (PHQ-9 score)	2.71±3.36	2.17±2.24	14.10±4.16	11.76±6.44
Major depressive disorder at baseline (MINI), n (%)	89 (3.1)	0	48 (41.0)	89 (100)
Use of antidepressants at baseline	187 (6.5)	146 (5.4)	35 (29.9)	26 (29.2)
**Diet**				
Energy intake (Kcal)	2,180 ± 604	2,180 ± 601	2,145 ± 643	2,218 ± 646
Protein total (g/day)	85.7 ± 23.0	85.8 ± 22.9	83.8 ± 26.3	84.1 ± 23.0
Carbohydrates total (g/day)	232.5 ± 69.5	232.3 ± 69.3	234.5 ± 72.6	242.8 ± 73.7
Fat total (g/day)	84.3 ± 31.1	84.3 ± 30.9	83.0 ± 32.6	85.9 ± 32.8
Fatty acids total saturated (g/day)	29.6 ± 12.0	29.6 ± 12.0	29.6 ± 12.0	29.6 ± 12.4
Fatty acids total monounsaturated (d/day)	29.7 ± 11.4	29.7 ± 11.3	29.7 ± 11.4	30.3 ± 11.8

Fatty acids total polyunsaturated (g/day)	17.7 ± 7.7	17.7 ± 7.7	17.7 ± 7.7	18.8 ± 8.6
Alcohol intake (g/day)	12. 2 ± 13.9	12.4 ± 13.9	9.1 ± 13.8	10.7 ± 15.9
DHD, (range 0–140)	83.6 ± 14.7	83.8 ± 14.7	80.1 ± 14.2	80.2 ± 14.2
Mediterranean Score, (range 0–9)	4.56 ± 1.64	4.57 ± 1.60	4.28 ± 1.63	4.42 ± 1.62
DASH score, (range 8–40)	24.0 ± 4.5	24.1 ± 4.5	23.0 ± 4.4	23.4 ± 4.1

* Results are presented as mean ± SD or n (%). MDD=Major Depressive Disorder.

Table S2. Cross-sectional association of dietary patterns with prevalent clinically relevant depressive symptoms and major depressive disorder.

**Clinical relevant depressive symptoms (PHQ-9 > 10)**	**Model 1 OR (95% CI) n = 112/2,666**	**Model 2 OR (95% CI) n = 111/2,637**	**Model 3 OR (95% CI) n = 101/2,483**
DHD-score			
Standardized score*	0.86 (0.70–1.06)	0.90 (0.73–1.11)	0.91 (0.72–1.16)
Tertiles			
Low (≤ 77.27) [n=49/912]	**Ref**	**Ref**	**Ref**
Medium (77.27–90.43) [n=36/914]	0.87 (0.55–1.39)	0.98 (0.61–1.57)	1.09 (0.66–1.81)
High (> 90.43) [n=32/914]	0.84 (0.51–1.37)	0.93 (0.56–1.55)	0.99 (0.56–1.75)
Linear trend p-value+	0.224	0.442	0.850
Mediterranean Diet Score			
Standardized score*	0.95 (0.78–1.15)	0.96 (0.79–1.18)	0.99 (0.80–1.24)
Cut-off**			
Low (0–3) [n=38/702]	**Ref**	**Ref**	**Ref**
Medium (4–6) [n=27/614]	0.83 (0.48–1.41)	0.85 (0.50–1.46)	0.73 (0.40–1.32)
High (6–9) [n=52/1424]	0.86 (0.55–1.34)	0.88 (0.56–1.39)	0.92 (0.57–1.49)
Linear trend p-value+	0.489	0.405	0.157
DASH score			
Standardized score*	0.92 (0.75–1.13)	0.97 (0.79–1.19)	1.04 (0.83–1.30)
Tertiles			
Low (≤ 22) [n=53/1022]	**Ref**	**Ref**	**Ref**
Medium (22–26) [n=36/909]	0.87 (0.55–1.37)	0.92 (0.58–1.46)	0.95 (0.58–1.56)
High (> 26) [n=28/809]	0.99 (0.61–1.62)	1.11 (0.67–1.83)	1.32 (0.77–2.27)
Linear trend p-value+	0.254	0.461	0.615
**Major depressive disorder (MINI)**	**Model 1 OR (95% CI) n = 85/2,693**	**Model 2 OR (95% CI) n = 85/2,663**	**Model 3 OR (95% CI) n = 75/2,509**
DHD-index			
Standardized score*	0.89 (0.70–1.12)	0.92 (0.73–1.17)	0.99 (0.75–1.30)
Tertiles			
Low (≤ 77.27) [n=37/924]	**Ref**	**Ref**	**Ref**
Medium (77.27–90.43) [n=29/921]	0.94 (0.56–1.58)	1.01 (0.59–1.70)	1.13 (0.64–1.99)
High (> 90.43) [n=23/923]	0.89 (0.50–1.58)	0.97 (0.54–1.73)	1.09 (0.56–2.14)
Linear trend p-value+	0.259	0.363	0.761
Mediterranean Diet Score			
Standardized score*	1.03 (0.83–1.30)	1.04 (0.82–1.30)	1.13 (0.87–1.45)
Cut-off*			
Low (0–3) [n=25/715]	**Ref**	**Ref**	**Ref**
Medium (4–6) [n=19/622]	0.95 (0.51–1.77)	0.92 (0.49–1.73)	0.87 (0.43–1.76)
High (6–9) [n=45/1431]	1.13 (0.68–1.90)	1.13 (0.67–1.90)	1.33 (0.76–2.35)
Linear trend p-value +	0.156	0.145	**0.044**
DASH score			

Standardized score*	1.00 (0.79–1.25)	1.04 (0.82–1.31)	1.09 (0.84–1.41)
Tertiles			
Low (≤ 22) [n=36/1039]	**Ref**	**Ref**	**Ref**
Medium (22-26) [n=28/917]	1.02 (0.61–1.71)	1.07 (0.63–1.80)	1.02 (0.58–1.80)
High (> 26) [n=25/812]	1.23 (0.71–2.12)	1.36 (0.78–2.37)	1.55 (0.85–2.85)
Linear trend p-value +	0.878	0.670	0.724

* Standard deviations for DHD, Mediterranean and DASH diet scores were 14.7, 1.64 and 4.5, respectively.
** Based on literature, Trichopoulou A. et al. (30).
+ Based on median.
Model 1 adjusted for socio-demographic characteristics (age, sex, level of education) and diabetes status.
Model 2 additional adjustment for cardiovascular risk factors: history of CVD, hypertension, total cholesterol and HDL cholesterol, waist circumference) and partner status.
Model 3 additional adjustment for lifestyle factors (MVPA, smoking, and energy intake).
(n cases/non cases), [n=cases/non-cases].

Table S3. Sensitivity analysis of the association of dietary patterns with incident clinically relevant depressive symptoms during 7-years of follow-up (median 6.1 years) additionally adjusted.

**Models**	**Incident Clinically Relevant Depressive Symptoms (PHQ-9 ≥ 10) HRs (95% CI)**		
**DHD-score**	Standardized score	Tertiles	
1) model 3+ alcohol intake (n= 280)	0.83 (0.73–0.96)	Low	Ref
		Medium	0.83 (0.63–1.12)
		High	0.77 (0.55–1.06)
2) model 3 + occupational status (n= 234 cases)	0.88 (0.76–1.01)	Low	Ref
		Medium	0.91 (0.67–1.25)
		High	0.83 (0.59–1.18)
3) model 3 + antidepressant drugs use (n= 280 cases)	0.89 (0.78–1.01)	Low	Ref
		Medium	0.88 (0.66–1.17)
		High	0.87 (0.63–1.20)
4) model 3 + baseline MDD (n= 280 cases)	**0.86 (0.75–0.98)**	Low	Ref
		Medium	0.88 (0.66–1.17)
		High	0.82 (0.60–1.13)
5) model 3 excluding antidepressant users (n= 239 cases)	0.88 (0.76–1.01)	Low	Ref
		Medium	0.82 (0.60–1.12)
		High	0.85 (0.60–1.19)
6) model 3 excluding baseline MDD (n= 264 cases)	**0.87 (0.76–0.99)**	Low	Ref
		Medium	0.85 (0.64–1.15)
		High	0.83 (0.60–1.14)
7) model 3 excluding lifetime MDD (n= 126 cases)	**0.73 (0.60–0.90)**	Low	Ref
		Medium	0.76 (0.50–1.17)
		High	0.70 (0.44–1.13)
8) model 3 excluding participants with maximum 2 missing PHQ-9 data over 7-years follow-up (n= 280 cases)	**0.88 (0.77–1.00)**	Low	Ref
		Medium	0.90 (0.68–1.20)
		High	0.81 (0.59–1.11)
**Mediterranean Diet Score**	Standardized score	Cut-off	
1) model 3+ alcohol intake (n= 280)	0.92 (0.81–1.05)	Low	Ref
		Medium	0.94 (0.68–1.31)
		High	0.96 (0.72–1.28)
2) model 3 + occupational status (n= 234 cases)	0.89 (0.78–1.02)	Low	Ref
		Medium	0.93 (0.67–1.29)
		High	0.93 (0.67–1.28)
3) model 3 + antidepressant drugs use (n= 280 cases)	0.92 (0.81–1.04)	Low	Ref
		Medium	1.03 (0.77–1.39)
		High	0.95 (0.70–1.28)

4) model 3 + baseline MDD (n= 280 cases)	0.90 (0.80–1.02)	Low	Ref
		Medium	0.94 (0.70–1.27)
		High	0.91 (0.67–1.22)
5) model 3 excluding antidepressant users (n= 239 cases)	0.90 (0.79–1.04)	Low	Ref
		Medium	0.96 (0.70–1.33)
		High	0.97 (0.70–1.33)
6) model 3 excluding baseline MDD (n= 264 cases)	0.90 (0.79–1.02)	Low	Ref
		Medium	0.99 (0.73–1.34)
		High	0.89 (0.65–1.22)
7) model 3 excluding lifetime MDD (n= 126 cases)	**0.79 (0.65–0.96)**	Low	Ref
		Medium	1.01 (0.65–1.56)
		High	0.96 (0.61–1.50)
8) model 3 excluding participants with maximum 2 missing PHQ-9 data over 7-years follow-up (n= 280 cases)	0.90 (0.80–1.02)	Low	Ref
		Medium	0.97 (0.72–1.31)
		High	0.89 (0.66–1.20)
**DASH score**	Standardized score	Tertiles	
1) model 3+ alcohol intake (n= 280)	0.95 (0.83–1.07)	Low	Ref
		Medium	1.06 (0.81–1.39)
		High	0.83 (0.60–1.15)
2) model 3 + occupational status (n= 234 cases)	0.91 (0.79–1.05)	Low	Ref
		Medium	0.97 (0.73–1.31)
		High	0.75 (0.52–1.07)
3) model 3 + antidepressant drugs use (n= 280 cases)	0.94 (0.83–1.07)	Low	Ref
		Medium	1.05 (0.80–1.38)
		High	0.85 (0.62–1.18)
4) model 3 + baseline MDD (n= 280 cases)	0.94 (0.83–1.07)	Low	Ref
		Medium	1.05 (0.80–1.38)
		High	0.82 (0.60–1.13)
5) model 3 excluding antidepressant users (n= 239 cases)	0.95 (0.83–1.09)	Low	Ref
		Medium	1.03 (0.77–1.38)
		High	0.86 (0.61–1.21)
6) model 3 excluding baseline MDD (n= 264 cases)	0.95 (0.84–1.09)	Low	Ref
		Medium	1.06 (0.80–1.40)
		High	0.83 (0.59–1.16)
7) model 3 excluding lifetime MDD (n= 126 cases)	0.83 (0.69–1.01)	Low	Ref
		Medium	1.19 (0.81–1.76)
		High	**0.56 (0.33–0.97)**
8) model 3 excluding participants with maximum 2 missing PHQ-9 data over 7-years follow-up (n= 280 cases)	0.95 (0.84–1.08)	Low	Ref
		Medium	1.07 (0.82–1.40)
		High	0.85 (0.61–1.17)

Model 3: age, sex, level of education, diabetes status, history of CVD, hypertension, total cholesterol and HDL cholesterol, waist circumference, partner status, MVPA, smoking, and energy intake.
